# Surfaceome and Proteosurfaceome in Parietal Monoderm Bacteria: Focus on Protein Cell-Surface Display

**DOI:** 10.3389/fmicb.2018.00100

**Published:** 2018-02-14

**Authors:** Mickaël Desvaux, Thomas Candela, Pascale Serror

**Affiliations:** ^1^Université Clermont Auvergne, INRA, UMR454 MEDiS, Clermont-Ferrand, France; ^2^EA4043 Unité Bactéries Pathogènes et Santé, Châtenay-Malabry, France; ^3^Micalis Institute, INRA, AgroParisTech, Université Paris-Saclay, Jouy-en-Josas, France

**Keywords:** Gram-positive bacteria, cell-surface protein, surface proteome, subcellular localization, pili/fimbriae/curli, lipoproteins, LPXTG sortase-dependent proteins, membrane proteins

## Abstract

The cell envelope of parietal monoderm bacteria (archetypal Gram-positive bacteria) is formed of a cytoplasmic membrane (CM) and a cell wall (CW). While the CM is composed of phospholipids, the CW is composed at least of peptidoglycan (PG) covalently linked to other biopolymers, such as teichoic acids, polysaccharides, and/or polyglutamate. Considering the CW is a porous structure with low selective permeability contrary to the CM, the bacterial cell surface hugs the molecular figure of the CW components as a well of the external side of the CM. While the surfaceome corresponds to the totality of the molecules found at the bacterial cell surface, the proteinaceous complement of the surfaceome is the proteosurfaceome. Once translocated across the CM, secreted proteins can either be released in the extracellular milieu or exposed at the cell surface by associating to the CM or the CW. Following the gene ontology (GO) for cellular components, cell-surface proteins at the CM can either be integral (GO: 0031226), i.e., the integral membrane proteins, or anchored to the membrane (GO: 0046658), i.e., the lipoproteins. At the CW (GO: 0009275), cell-surface proteins can be covalently bound, i.e., the LPXTG-proteins, or bound through weak interactions to the PG or wall polysaccharides, i.e., the cell wall binding proteins. Besides monopolypeptides, some proteins can associate to each other to form supramolecular protein structures of high molecular weight, namely the S-layer, pili, flagella, and cellulosomes. After reviewing the cell envelope components and the different molecular mechanisms involved in protein attachment to the cell envelope, perspectives in investigating the proteosurfaceome in parietal monoderm bacteria are further discussed.

## Introduction

As the interface of the cell with its surrounding, the bacterial cell surface plays a crucial role in all types of interactions. In the first instance, the diversity of the bacterial cell envelope is generally viewed as dichotomic, on the one hand, the Gram-positive bacteria, and on the other hand, the Gram-negative bacteria (Desvaux et al., 2004, 2009). This difference is based on the result of the Gram staining method originally developed by the Danish pharmacologist and physician Hans Christian Joachim Gram (Gram, 1884) and still routinely used worldwide to differentiate bacteria (Beveridge, 2001). With the development of microscopic techniques, it first appeared the difference in staining was the result of profound divergence in structural organisation of the bacterial cell envelope, where Gram-positive bacteria have a thick cell wall (CW) sitting atop of a cytoplasmic membrane (CM) (Silhavy et al., 2010). Later on, molecular analyses further revealed that Gram-positive bacteria corresponded to a phylogenetically coherent group within the domain Bacteria and belonged to only two phyla, namely the low G+C% Gram-positive bacteria of the phylum Firmicutes and the high G+C% Gram-positive bacteria of the phylum Actinobacteria (Woese, 1987; Woese et al., 1990). Over the years, though, it appears this terminology presents some ambiguity when considering the diversity of the domain Bacteria (Desvaux et al., 2009). Considering the term “Gram-positive bacteria,” it can refer to three distinct, and sometimes incompatible elements, i.e., a Gram staining result, a cell envelope architecture and/or a taxonomic group. For instance, bacteria of the class Mollicutes, comprising the genus *Mycoplasma*, cannot retain the Gram stain because they naturally lack a CW although the low G+C% content of their genomes and other molecular markers resemble those of Gram-positive bacteria of the phylum Firmicutes (Razin et al., 1998). Species of the genus Mycobacterium possess a peculiar cell envelope with a mycomembrane preventing Gram staining and thus require alternative staining methods called acid-fast (Somoskovi et al., 2001) but nonetheless belong to the high G+C% Gram-positive bacteria of the phylum Actinobacteria (Draper, 1998). In some deep branches of the phylum Firmicutes, some bacteria clearly exhibit Gram-negative cell envelope for which a new class was proposed, i.e., the Negativicutes (Marchandin et al., 2010).

Inspired by the research work of Gupta (1998a,b, 2000), the description of the bacterial cell envelope respective to the number of biological membranes appeared much more definite and was first reintroduced in the field of bacterial protein secretion (Desvaux et al., 2009). While monoderm bacteria refer to species exhibiting only one biological membrane, namely the CM, diderm bacteria correspond to species exhibiting two biological membranes, i.e., an inner membrane and an outer membrane. Monoderm bacteria can be further discriminated into (i) simple monoderm, lacking a CW (e.g., bacteria from the genus *Mycoplasma*), and (ii) parietal monoderm, exhibiting a CW (archetypal Gram-positive bacteria) (Sutcliffe, 2010; Gupta, 2011). As such, parietal monoderm bacteria include most Firmicutes, e.g., from the class Bacilli and Clostridia, but of course exclude the class Mollicutes and Negativicutes as well as the Actinobacteria exhibiting a mycolate outer membrane.

**FIGURE 1:**
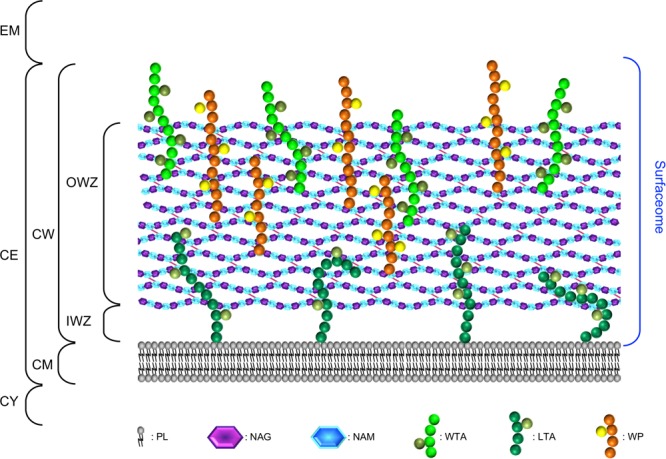
The surfaceome of parietal monoderm bacteria with respect of the organisation and composition of the cell envelope. The cell envelope (CE) of parietal monoderm bacteria is composed of a biological membrane acting as selective permeable barrier, i.e., the cytoplasmic membrane (CM) and a cell wall (CW) providing some resistance to mechanical stresses (e.g., internal turgor pressure) but also somehow acting as a philtre. While the CM is composed of phospholipids (PLs), the CW can be further subdivided into the inner wall zone (IWZ) and the outer wall zone (OWZ). The OWZ constitutes the main CW fabric. It is composed of *N*-acetylglucosamine (NAG) and *N*-acetylmuramic acid (NAM), both constituting the peptidoglycan (PG) with which wall teichoic acids (WTAs), and wall polysaccharides (WPs) are anchored. Lipoteichoic acids (LTAs) are anchored to the CM and protrude from the CM. As revealed by electron microscopy studies and contrary to the OWZ, the IWZ is a thinner zone of low density most certainly devoid of most cross-linked polymeric CW network, except LTAs and some proteins, e.g., lipoproteins (Matias and Beveridge, 2005, 2006); because this zone is not strictly bounded by two biological membranes like in diderm bacteria, the IWZ resembles but cannot be considered as a periplasm *sensu stricto*, i.e., it presents some analogies but no homology (Buist et al., 2008; Chagnot et al., 2013). In addition to the proteins present both at the CM and CW and that are not depicted here for clarity (see text and **Figures 4–6**), these different macromolecular molecules exposed on the external side of the CM constitute the surfaceome in parietal monoderm bacteria. CY, cytoplasm; EM, extracellular milieu.

**FIGURE 2:**
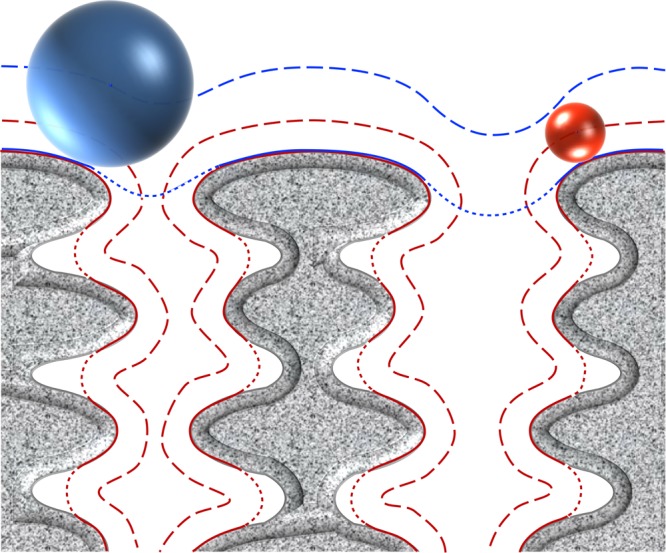
Concepts of molecular surface, contact surface, accessible surface, and reentrant surface to define the bacterial cell surface in parietal monoderm bacteria. Taking molecules of different sizes, their penetration in the cell envelope differs. The blue sphere represents a molecule of high molecular weight unable to penetrate the CW fabric (depicted in grey), whereas the red sphere represents a smaller molecule diffusing through. Depending on the molecules considered, the definition of the bacterial cell surface will also differ. The continuous lines represent the contact surface that is the molecular surface that actually comes in direct contact with the surface of the molecule considered. The dashed lines represent the accessible surface that is the continuous sheet referring to the centre of the molecule considered. The dotted lines correspond to the reentrant surface that is the interior-facing part of the molecule considered when it cannot come in direct contact with the molecular surface of the cell envelope. The definition of bacterial cell surface of parietal monoderm bacteria is thus very different when referring to the molecular surface of the cell envelope or the contact, accessible and reentrant surfaces with respect of the size of the molecule under consideration.

The CW of parietal monoderm bacteria is a complex structure composed at least of peptidoglycan (PG) covalently linked to other biopolymers, such as teichoic acids, polysaccharides, polyglutamate, or proteins (Shockman and Barrett, 1983; **Figure 1**). While constituting the outermost layer of the cell envelope of parietal monoderm bacteria, the CW is not impermeable but on the contrary a porous and penetrable structure. As such, cell envelope proteins are in contact with the external environment without ever having a domain protruding out the confines of the CW. Like for the fractal dimension of the protein surface (Richards, 1977; Banerji and Navare, 2013), the nature and definition of the bacterial cell surface strictly depends on the molecule considered, e.g., a water molecule or a globular protein, which can enter in contact, access, diffuse or penetrate differently the CW (**Figure 2**). To be exposed at the cell surface of parietal monoderm bacteria, proteins need to be first secreted across the CM. Several secretion systems allow protein translocation in parietal monoderm bacteria (Tjalsma et al., 2004; Desvaux et al., 2005; Desvaux and Hébraud, 2006; Sibbald et al., 2006; Chagnot et al., 2013), namely (i) the Sec (secretion), (ii) the Tat (twin-arginine translocation), (iii) ABC protein exporter, (iv) the FPE (fimbrilin-protein exporter), (v) the FEA (flagella export apparatus), and (vi) the ESX (ESAT-6 system), also called Wss (WXG100 secretion system). Of note, the status of the holins (hole forming) as protein secretion systems *per se* remain controversial (Desvaux, 2012). Proteins secreted via the Sec translocon generally possess a targeting signal called the signal peptide (SP) of type I (SP I), which is composed of three non-conserved domains, namely the n-domain (positively charged and at the N-terminus), the h-domain (a-helical hydrophobic core region), and the c-domain (cleavage site processed by a membrane-bound signal peptidase) (Fekkes and Driessen, 1999). While proteins secreted via Sec, Tat ABC exporter and FPE possess N-terminal SPs with some specificities, the signal targeting proteins to the FEA or ESX remain elusive. Besides transport across the CM, the transport and maturation of secreted proteins across the CW can be regulated by different mechanisms, such as the proteolytic maturation of secreted proenzymes, the requirement of divalent cations for activation or the post-translocational intervention of peptidyl-prolyl isomerase chaperones (Forster and Marquis, 2012).

To explicitly describe the subcellular localization of proteins, the gene ontology (GO) respective to the cellular component is extremely useful (Ashburner et al., 2000; Chagnot et al., 2013). Indeed, secreted proteins can have different fate; they are either (i) associated to the CM (GO: 0005886), (ii) anchored to the CW (GO: 0009275), (iii) released in the extracellular milieu (GO: 0005576), the so-called exoproteins (extracellular proteins), or even (iv) injected into a prokaryotic or eukaryotic host cell. At the CM, proteins can be either integral (GO: 0031226), i.e., the IMPs (integral membrane proteins), or anchored to the membrane (GO: 0046658), i.e., the lipoproteins. At the CW, proteins can be covalently bound, i.e., the LPXTG-proteins, or bound through weak interactions, i.e., the CW binding proteins. It is worth stressing that all these extracytoplasmic proteins located at the cell envelope, wherever at the CM or the CW, can be considered as surface exposed. Besides monopolypeptides, some organelles can also be present and result from the assembly of protein subunits to form supramolecular structures, such as the well-known pili and flagella, but also the S-layer or cellulosome in some bacterial species.

Following the etymological meaning of the Greek suffix “-ome” (Lederberg and McCray, 2001), the totality of the molecules found at the bacterial cell surface corresponds to the surfaceome. Because of the spongy structure of the CW, it is misleading to restrict the surface of parietal monoderm bacteria to molecules strictly displayed at the outermost molecular layer of the CW. Instead, the cell surface of a parietal monoderm bacterium fits tightly to the molecular outline of the CW components and to the external side of the CM (**Figure 2**); as a biological membrane, the CM has a selective permeability contrary to the CW. The CW is not a rigid shell but constitutes a matrix, forming an elastic polyelectrolyte gel (Doyle and Marquis, 1994; Neuhaus and Baddiley, 2003), which would then acts like a sieve during the dynamic transit of solutes. The proteosurfaceome is the proteinaceous subset of the surfaceome found at the CW and totally or partially exposed on the external side of the CM.

## The Surfaceome of Parietal Monoderm Bacteria

The cell envelope of parietal monoderm bacteria is composed of a CM and a CW, which can be divided into the inner wall zone (IWZ) and outer wall zone (OWZ) (Merchante et al., 1995; Matias and Beveridge, 2005; Zuber et al., 2006; **Figure 1**). The CW surrounding the CM is made of lipoteichoic acids (LTAs) and a thick layer of PG, decorated with wall teichoic acids (WTAs), wall polysaccharides (WPs), or/and polyglutamate. The CW also accommodates some proteins, including monopolypeptides and cell-surface supramolecular protein structures, namely pili, flagella, cellulosome, S-layer. Altogether these different macromolecular molecules and associated molecules constitute the surfaceome. This part focuses on the components of the cell envelope, excluding the proteinaceous compounds discussed in the subsequent part. Cell envelope proteins actually interact with some of these components for anchoring via different molecular mechanisms.

### Composition and Organisation of the Cytoplasmic Membrane

The phospholipid bilayer of the membrane parietal monoderm bacteria is ∼90 Å thick and is composed of 10–40% lipids, 40–75% proteins, and 0.2–20% carbohydrates (Salton, 1967; Ghosh and Carroll, 1968; Bodman and Welker, 1969; Duda et al., 2006). Although membrane phospholipids vary from one species to another, the most commonly found in the CM are glycerophospholipids including phosphatidylglycerol, diphosphatidylglycerol (cardiolipin), and to some extend phosphatidylethanolamine and their amino acylated forms (Fischer et al., 1978; Roy, 2009; Malanovic and Lohner, 2016). Phospholipids vary also by their two fatty acid moieties, which impact on membrane fluidity (Mishra et al., 2012; Custer et al., 2014; Diomande et al., 2015; Malanovic and Lohner, 2016). Polyisoprenoid lipids are other important regulators of membrane fluidity. They constitute, together with cardiolipins and bacterial flotillins acting as scaffolding proteins, nanoscale functional membrane microdomains, which seem essential to the proper functioning of signal transduction cascades and protein transport in *Bacillus subtilis* and *Staphylococcus aureus* cells (Lopez and Kolter, 2010; Bramkamp and Lopez, 2015). By analogy with eukaryotic membranes, these microdomains are also referred to as lipid rafts. Consistently, membrane proteins or associated complexes constitute discrete focal sites in the CM and CW (Campo et al., 2004; Rosch et al., 2007; Lopez and Kolter, 2010; Kandaswamy et al., 2013). Biological significance of functional membrane microdomains could be to serve as platforms that control the assembly of membrane and CW proteins and multiprotein complexes involved in numerous cellular processes, such as cell division, protein trafficking, genetic transfer, or signal transduction (Lopez and Kolter, 2010; Schneider et al., 2015). Subcellular localization and spatiotemporal distribution of CM and CW proteins or supramolecular protein complexes are often intimately linked to their function and vary with the environmental conditions (Bierne and Dramsi, 2012; Mitra et al., 2016).

### Composition and Organisation of the Cell Wall

**FIGURE 3:**
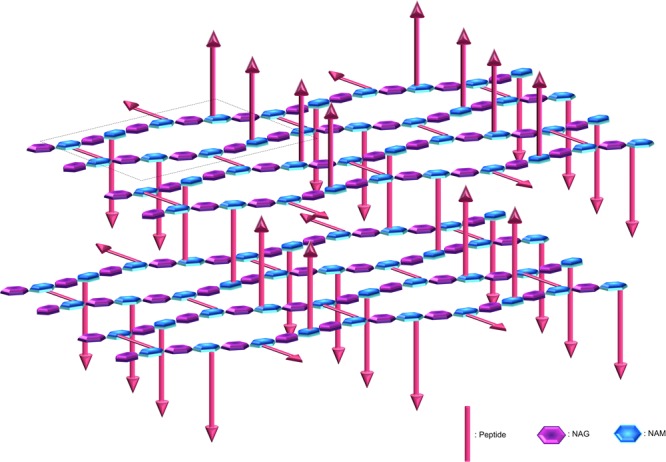
Peptidoglycan organisation at the cell wall. The peptidoglycan is composed of *N*-acetylglucosamine (NAG) and *N*-acetylmuramic acid (NAM) linked by β-1,4 bonds, where the NAM are further crosslinked via octapeptides either at the same plane or with the upper or lower layer (arrows represent peptides protruding up or down). The peptidoglycan is tiled with hexagonal tesserae, which constitute the structural unit of the CW fabric (one basic unit constituting a tessera is displayed inside the dotted frame). Two layers of tesserae are here schematically represented to highlight the network form by the peptide crosslinking. Of note, defects due to abnormal tesserae with more edges and larger area can also occur and resulting in the increase in porosity.

The OWZ constitutes the main part of the CW. It is 15–30 nm thick and comprises the PG and WTA polymers (Navarre and Schneewind, 1999; Vollmer et al., 2008). The PG is made of *N*-acetylglucosamine (NAG) and *N*-acetylmuramic acid (NAM) forming disaccharide glycan chains of various lengths that are cross-linked by peptides. PG composition depends on bacteria (Schleifer and Kandler, 1972). The glycan chain is uniform, whereas the peptide moiety and the cross-links are variable. The two major PGs in parietal monoderm bacteria have a meso-diaminopimelic acid (A2pm) or a lysin at the third position of the peptide. At this position, the cross-link occurs directly or through a penta-glycine bond, respectively. In *B. subtilis*, it is estimated that the glycan chain length is 1300 disaccharides in average, and that approximately 20% of the peptide chains are cross-linked (Ward, 1973; Atrih et al., 1998; Hayhurst et al., 2008). These glycan chains form helices of ∼50 nm width, and it was proposed that these cable-like structures coil around the narrow axis of the bacterium and are cross-linked by peptides (Hayhurst et al., 2008). The glycan chains of ovococcal bacteria, e.g., *Streptococcus* sp., are formed of more than 100 disaccharide units in average, whereas the glycan chains of cocci, e.g., *Staphylococcus* sp., are relatively short with 5–10 disaccharide units in average (Wheeler et al., 2011). The average effective mesh size in PG, i.e., the tessera, is estimated at 2.2 nm (Koch, 1990; Demchick and Koch, 1996; **Figure 3**). In other words, hydrophilic molecules of about 25 kDa (but also probably up to 50 kDa) can freely pass through a structured CW meshwork. Along with this, the CW network is actually not perfect, e.g., pseudo-tessera, and numerous PG defects cause increase in the porosity (Pink et al., 2000; Turner et al., 2013; Kim et al., 2015). Of note, though, the critical hole size in the CW beyond which lysis occurs, is estimated in the range of 15–24 nm (Mitchell et al., 2013). The OWZ of parietal monoderm bacteria is a very dynamic structure, as bacterial growth requires constant remodelling of the CW, which has a turnover rate of 50% per generation (Koch and Doyle, 1985). Remodelling is mediated by CW-anchored autolysins that are active on the outermost layer of the PG (Jolliffe et al., 1981).

LTAs and WTAs are zwitterionic polymers anchored to the CM and CW, respectively. They are major polyanionic teichoic acids of the envelope of parietal monoderm bacteria. LTAs are localised in the IWZ at the interface of the CM and the CW (Neuhaus and Baddiley, 2003; Reichmann and Grundling, 2011; Schneewind and Missiakas, 2012; Percy and Grundling, 2014). The most common LTA structure found in Firmicutes and, referred as type I LTAs, consists in a polyglycerol phosphate polymer linked to a glycolipid anchor, often a diglucosyl-diacylglycerol (Glc2-DAG), anchored to the CM. Type II, III, IV, and V LTAs have more complex repeating units that contain glycosyl residues, e.g., in *Streptococcus pneumoniae*, type IV LTA is decorated with phosphocholine.

WTAs are covalently attached by the PG disaccharide unit via a phosphodiester linkage to NAM (Neuhaus and Baddiley, 2003; Brown et al., 2013). Although the structures of WTAs vary considerably between species, the most common ones are composed of glycerol-phosphate or ribitol-phosphate repeats. LTAs and WTAs are often modified with sugar moieties and D-alanine esters, which introduce positive charges to neutralise the negatively charged phosphates in the polymer backbone (Wooldridge and Williams, 1993; Xia et al., 2010; Schneewind and Missiakas, 2012; Percy and Grundling, 2014; Carvalho et al., 2015). In addition to their diversity between and within species, the degree of D-alanylation of teichoic acids is fine tuned in changing environments and thus likely influences the protein repertoire displayed at the CW. The zwitterionic WTA polymers potentially contribute to the sequestration of divalent cations within the OWZ, including Ca^2+^, Mg^2+^, and Fe^2+^ (Beveridge and Murray, 1980), and might thus influence the regulation of protein transport across the CW (Forster and Marquis, 2012).

WPs have various compositions, e.g., teichuronic acids in *Bacillus* (Ward, 1981) or highly diverse heteropolysaccharides in *Lactococcus* (Yasuda et al., 2011; Vinogradov et al., 2013; Ainsworth et al., 2014), which complexity and diversity can be even greater than expected as revealed by the ever increasing genome data regularly made available. The last and most external layer of the CW may be composed of a capsule, generally composed of WPs (Jones, 2005; Yother, 2011). Although the WP capsule structures are well documented, the anchoring was recently proposed to be at the β-D-*N*-acetylglucosamine of the PG via a direct glycosidic bond (Larson and Yother, 2017). In some cases, the capsule is composed of polyglutamate, e.g., in *Bacillus anthracis* (McLean et al., 1992; Candela and Fouet, 2006). Poly-γ-D-glutamate anchoring was reported to be covalent at the PG (Candela and Fouet, 2005; Candela et al., 2005). However, the exact anchoring mechanism is still controversial and may be either on the A2pm or on the PG glucosamine (Richter et al., 2009; Candela et al., 2014).

Overall, the CW of parietal monoderm bacteria is a complex structure that protects them from mechanical and osmotic lysis, and serves as a scaffold for anchoring proteins, glycopolymers, and cations that perform various functions (Navarre and Schneewind, 1999; Weidenmaier and Peschel, 2008). While WPs or WTAs can be essential for bacterial growth (Oh et al., 2017), WTAs have been shown to be dispensable in some other bacterial species (Chapot-Chartier and Kulakauskas, 2014; Mistou et al., 2016). However, wall rhamnose polysaccharides (RhaWPs) can be a functional counterpart of WTAs, as suggested in *Streptococcus agalactiae* and *Streptococcus pyogenes* (Caliot et al., 2012; van Sorge et al., 2014), where they appear to be covalently linked to PG NAM (Deng et al., 2000).

## Cell-Surface Proteins Localised at the Cytoplasmic Membrane (Go: 0005737)

Cell-surface proteins specifically localised at the CM can either be integral to the CM (GO:0031226) or anchored to the CM (GO: 0046658). Besides, some proteins can interact by weak interactions with components of the CM surface and be extrinsic to the CM (GO:0019897).

### Proteins Integral to the Cytoplasmic Membrane (GO: 0031226): The IMPs

**FIGURE 4:**
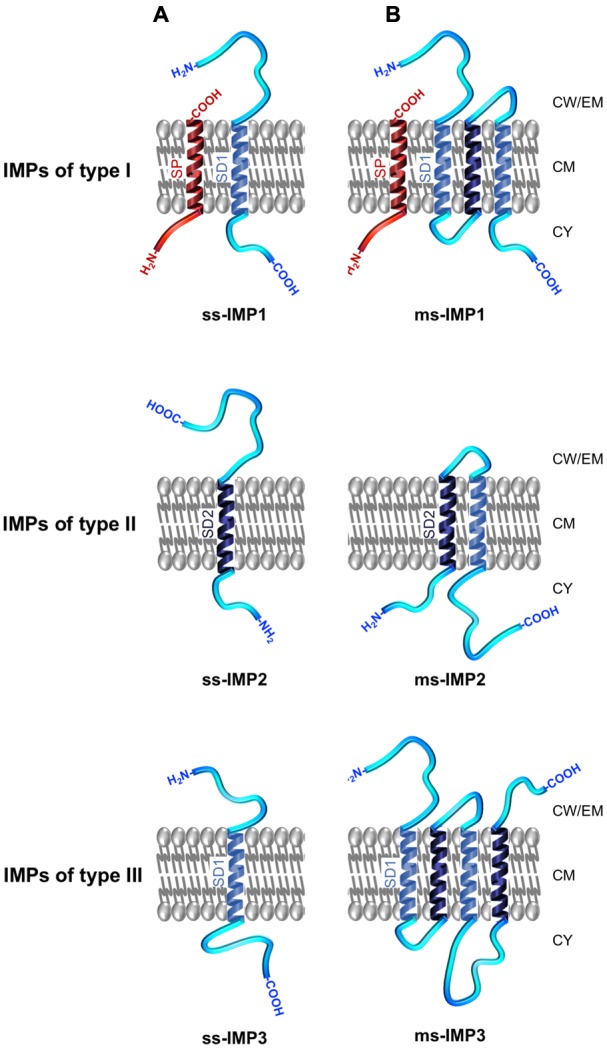
Topology and nomenclature of IMPs. IMPs are primarily categorised into **(A)** single-spanning IMPs (ss-IMPs) and **(B)** multi-spanning IMPs (ms-IMPs). Indeed, IMPs are anchored to the CM via hydrophobic transmembrane α-helical peptide domains (TMDs); when a TMD has a N_out_–C_in_ topology, it is called a signal domain of type I (SD1; depicted in light blue), whereas a TMD with N_in_–C_out_ topology is called a signal domain of type II (SD2; depicted dark blue) (White and von Heijne, 2004). In ss-IMPs, only one TMD is present, whereas at least two TMDs are present in ms-IMPs. Whenever ss-IMPs or ms-IMPs, they are further subcategorised into three types. A ss-IMP of type I (ss-IMP1) possesses a cleavable N-terminal signal peptide (SP; depicted in red) and are actually anchored to the CM by a SD1 (TMD with a C_in_–N_out_ topology). A ss-IMP of type II (ss-IMP2) is anchored to the CM by a SD2 (TMD with a N_in_–C_out_ topology). Like for a ss-IMP1, a ss-IMP of type III (ss-IMP3) is anchored to the CM by a SD1 but it did not originally exhibit a SP. For ms-IMPs, the classification is similar and based on the most N-terminal TMD anchoring the ms-IMP to the CM. As such, a ms-IMP of type I (ms-IMP1) has a cleavable SP followed by a SD1. A ms-IMP of type II (ms-IMP2) has a SD2 as the most N-terminal TMD. A ms-IMP of type III (ms-IMP3) has a SD1 as the most N-terminal TMD (and no cleavable SP). Of note, the TMD of a cleavable SP actually corresponds to a SD2; as such, a SD2 in IMPs of type II can be referred as an uncleavable SP. In ms-IMPs, a SD1 necessarily alternates with a SD2 along the polypeptide chain, and vice versa. Except for the TMDs, other regions of the IMPs can be in contact with the IWZ but also the OWZ or the extracellular milieu.

As a common theme, all IMPs exhibit hydrophobic transmembrane α-helical domains (TMDs) enabling anchoring of the protein to the membrane (White and von Heijne, 2004). IMPs can be broadly discriminated between single-spanning IMPs (ss-IMPs) exhibiting a single TMD and multispanning-IMPs (ms-IMPs) with more than one TMD (**Figure 4**; Goder and Spiess, 2001; Higy et al., 2004). Whereas most IMPs are not synthesised with a cleavable N-terminal SP, some IMPs are (Facey and Kuhn, 2004). For the latters and after cleavage of the hydrophobic transmembrane α-helical SP by a signal peptidase (SPase), the ss-IMPs remain anchored to CM thanks to an additional hydrophobic TMD, i.e., the stop-transfer sequence also called signal domain of type I (SD1), which exhibits a N_out_–C_in_ topology; as such, these ss-IMPs refer to the type I (ss-IMP1; **Figure 4**). Type II ss-IMPs (ss-IMP2) have a signal-anchor sequence also called signal domain of type II (SD2), with a N_in_–C_out_ topology, which actually corresponds to an uncleavable SP. Type III ss-IMPs (ss-IMP3) have reverse signal-anchor sequence, i.e., a SD1 (TMD with a N_out_–C_in_ topology); in the literature, they are sometimes described as ss-IMP1 without SP since the reverse signal-anchor sequence is a SD1. Of note, while the translocation mechanism of both type I and type II IMPs is in line with our current knowledge about the Sec/YidC translocation, i.e., involving an N-terminal SP (whenever cleavable or uncleavable) targeting the protein to CM, the mechanism for the translocation of type III IMPs in the absence of a SP remain unclear. In ms-IMPs, the type I (N_out_–C_in_ TMD topology) and type II (N_in_–C_out_ TMD topology) signals alternate along the protein sequence. Based on topology of the most N-terminal TMD enabling anchoring of the ms-IMP to the CM, the three types mentioned here above can be discriminated (**Figure 4**).

IMP biogenesis in lipopolysaccharidic-diderm bacteria (archetypal Gram-negative bacteria) involves an integrase known as YidC (Scotti et al., 2000). Up to two paralogues of the integrase YidC have been uncovered in parietal monoderm bacteria, namely SpoIIIJ and YqjG (Tjalsma et al., 2000; van Wely et al., 2001). While both SpoIIIJ and YqjG are involved IMP biogenesis and are essential for cell viability (Murakami et al., 2002; Tjalsma et al., 2003), SpoIIIJ is required for sporulation in *B. subtilis* but not YqjG (Errington et al., 1992; Murakami et al., 2002). Lately, these proteins have been renamed YidC1 and YidC2, respectively, in parietal monoderm bacteria (Funes et al., 2009; Wang and Dalbey, 2011; Palmer et al., 2012). In *E. coli*, YidC is associated to the Sec translocase enabling insertion of all IMPs to the CM in a SRP (signal-recognition particle) dependent mechanism (Scotti et al., 2000; Fröderberg et al., 2003; Ziehe et al., 2017). In this species, the YidC pathway is quite versatile since integration of IMPs to the CM can be SecA-, SecB-, and/or Sec-independent (Samuelson et al., 2000; Beck et al., 2001; Yen et al., 2002; Fröderberg et al., 2003; White and von Heijne, 2004). Moreover, flotillin-like proteins could contribute to the insertion of IMPs (Dempwolff et al., 2016). So far, these aspects have been poorly investigated in parietal monoderm bacteria.

### Cell-Surface Proteins Anchored to the Cytoplasmic Membrane (GO: 0046658): The Lipoproteins

**FIGURE 5:**
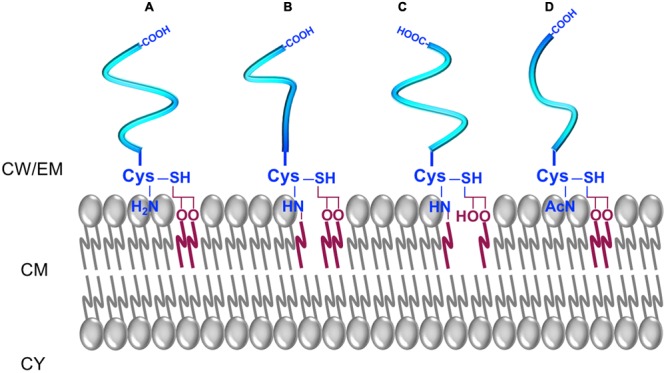
The different forms of lipoproteins in parietal monoderm bacteria. **(A)** A diacyl-lipoprotein contains an *N*-acyl-*S*-diacylated cysteine residue. **(B)** A *N*-acylated-triacyl-lipoprotein contains an *N*-acyl-*S*-triacylated cysteine residue. **(C)** A lyso-lipoprotein contains an *N*-acyl-*S*-monoacyl-glyceryl-cysteine. **(D)** A *N*-acetyl-form contains a *N*-acetyl-*S*-diacyl-glyceryl-cysteine.

In parietal monoderm bacteria, lipoproteins are synthesised as pre-prolipoproteins that are exported by the Sec pathway and exposed on the outer face of the CM (Hutchings et al., 2009; **Figure 5**). The pre-prolipoproteins exhibit a SP of type II (SP II) that is harbouring a conserved lipobox motif at the cleavage site (Sutcliffe and Harrington, 2002). The consensus sequence for the lipobox is [LVI]_-3_-[ASTVI]_-2_-[GAS]_-1_-[C]_+1_ (Sutcliffe and Harrington, 2002; Babu et al., 2006). Once translocated across the CM, the lipoprotein maturation pathway in parietal monoderm bacteria is a two-step process. First, the lipobox motif is recognised by a prolipoprotein diacylglyceryl transferase (Lgt), which transfers of a diacylglyceryl moiety from a glycerophospholipid onto the thiol group of the conserved cysteine, giving rise to the prolipoprotein. Then, the SP II of the prolipoprotein is cleaved off by a lipoprotein signal peptidase (Lsp), generating a mature lipoprotein. The lipoprotein is consequently covalently bound to the acyl moiety of two fatty acids from the diacylglyceride by a cysteine residue at position 1 of the N-terminal end (Lai et al., 1980). Besides this classical form of lipid-modified cysteine for lipoprotein anchoring to the CM, intensive mass spectrometry analyses revealed three novel forms of mature lipoproteins in parietal monoderm bacteria (Nakayama et al., 2012; **Figure 5**). The N-acylated triacyl form of lipoproteins containing *N*-acyl-*S*-diacyl-glyceryl-cysteine was identified in *S. aureus* and *S. epidermidis* (Kurokawa et al., 2009; Asanuma et al., 2011). The *N*-acetyl form of lipoproteins identified in different Bacillaceae contains *N*-acetyl-*S*-diacyl-glyceryl-cysteine (Kurokawa et al., 2012b). The lyso-form of lipoproteins containing an *N*-acyl-*S*-monoacyl-glyceryl-cysteine was identified in *Bacillus cereus, Enterococcus faecalis, Lactobacillus bulgaricus*, and *Streptococcus sanguinis* (Asanuma et al., 2011). It further appeared that environmental conditions influenced the ratio between diacyl and triacyl forms of lipoproteins in *S. aureus*, with an accumulation of the diacyl lipoprotein form at high temperatures and high salt concentrations (Kurokawa et al., 2012a). Together, these recent findings are suggestive of uncharacterised non-canonical pathways for differential lipoprotein lipidation in parietal monoderm bacteria, analogous to the N-acylation of the lipidated cysteine by the apolipoprotein *N*-acyltransferase (Lnt) in lipopolysaccharidic-diderm bacteria. Actually, the lipoprotein intramolecular transacylase (Lit) involved in N-lyso-form biosynthesis was recently identified in *E. faecalis* and *B. cereus* (Armbruster and Meredith, 2017). If N-acylation is likely to involve acyltransferases adapted to specific phospholipids as acyl-donor substrates, novel enzymes and maybe pathways are to be discovered to explain how these alternative *N*-acetyl lipoprotein forms are biosynthesised in parietal monoderm bacteria.

## Cell-Surface Proteins Localised at the Cell Wall (Go: 0009275)

**FIGURE 6:**
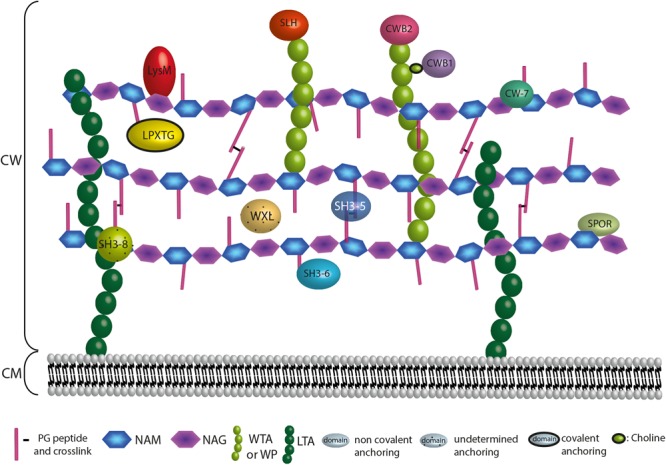
Anchoring localization of protein domains interacting with the CW. The localization of the CW proteins depends on their domains. Domains are interacting covalently or not at the bacterial CW through interaction with surface structures that are the PG, the WTAs, the WPs or the LTAs. LPXTG proteins are covalently attached to the A2pm or K residue of the PG. Proteins harbouring a LysM, SH3 of type 5 (SH3-5), SH3 of type 6 (SH3-6), SPOR, or CW-7 domain interact non-covalently with the PG. WXL interacts with PG but the precise anchoring region is undetermined. Proteins possessing a CWB2 or SLH domain are localised at the WTAs or WPs extremities, whereas proteins harbouring a CWB1 domain interact with the WTAs through a choline. For SH3 of type 8 (SH3-8), the CW target remains controversial.

The first surface associated proteins were described because of their activities on the bacterial CW. Most of them were autolysins or proteases. PG-binding domains were thereafter observed thanks to the sequencing data and bioinformatic analyses. Indeed, amino acid repetitions involved in the surface binding were highlighted. Most of the characterised and conserved domains are registered and classified by bioinformatic resources, especially InterPro (IPR; Zdobnov and Apweiler, 2001; Finn et al., 2017) regrouping several databases for motif signatures, such as Pfam (Soohammer et al., 1997; Finn et al., 2016), Prosite (Hulo et al., 2006; Sigrist et al., 2013), or SMART (Schultz et al., 1998; Letunic et al., 2015) (**Table 1**). Of note, the use of underscore (“_”), as given in the name of domains in databases, must be abstained by reminding the readers this sign is primarily designed for bioinformatics purpose when a space cannot be used due to command line constraints but are meant to be replaced by a space (“ ”) or a dash (“-”) in textbook. These binding domains allow protein subcellular location at the CW and are therefore often crucial for their activity on the surface structure and organisation (**Figure 6**). They can be divided into three main classes: domains that are (i) covalently attached to the PG, (ii) non-covalently bound to the PG, and (iii) non-covalently bound to WPs (**Figure 6**). Besides, the CW components targeted by some domains remain uncertain. These proteins are generally secreted by the Sec translocon and possess a SP I.

**Table 1:** Domains involved in protein attachment to the cell wall in parietal monoderm bacteria.

Name	Abbreviation	Other names^a^	InterPro	Other databases^b^	PDB^c^	CW ligand^d^
**Domain involved in covalent attachment to the CW**		
LPXTG domain	LPXTG		IPR019948	PF00746, PS50847, PR00015	3UXF	PG
**Domain involved in non-covalent attachment to the CW**		
Lysin motif	LysM		IPR018392	PF01476, SM00257, CD00118, PS51782, SSF54106	2MKX	PG
WXL domain	WXL		IPR027994	PF13731		PG
SH3 domain of type 3	SH3-3	SH3b	IPR003646	PF08239, SM00287, PS51781	4KRT	PG
SH3 domain of type 5	SH3-5		IPR003646	PF08460	5D76	PG
SH3 domain of type 6	SH3-6	SH3b1		PF12913	3M1U	PG
SH3 domain of type 7	SH3-7	SH3b2	IPR026864	PF12914	3M1U	PG
SH3 domain of type 8	SH3-8	GW	IPR025987	PF13457, PS51780	1M9S	PG and/or LTAs
Sporulation-related domain	SPOR		IPR007730	PF05036, PS51724, SSF110997	1X60	PG
Cell wall binding repeat of Cpl-7	CW-7		IPR013168	SM01095, PF08230	4CVD	PG
Peptidoglycan-binding domain of type 1	PGB1		IPR002477	PF01471, SSF47090	4XXT	PG
Peptidoglycan-binding domain of type 2	PGB2		IPR014927	PF08823		n.d.
Cell wall binding repeat of type 1	CWB1	ChBD	IPR018337	PF01473, PS51170	1HCX	Choline residues
Cell wall binding repeat of type 2	CWB2		IPR007253	PF04122		WPs
S-layer homology domain (SLH)	SLH		IPR001119	PF00395, PS51272	3PYW	PG
Clostridial hydrophobic repeat (ChW)	ChW		IPR006637	PF07538, SM00728		n.d.

*^*a*^Other names found in the literature to name the respective domain. Name and abbreviation given in the two first columns are preferred and must be favoured. SH3b, sarcome homology 3 domain of bacterial type; ChBD, choline-binding domain.*

*^*b*^Signatures from InterPro member databases used to construct the entry, namely from Pfam (PF), SMART (SM), Conserved Domain Database (CD), Prosite (PS), Prints (PR), SuperFamily (SF).*

*^*c*^Accession number to the resolved structure in PDB (protein data bank).*

*^*d*^CW, cell wall; PG, peptidoglycan; LTAs, lipoteichoic acids; WTAs, wall teichoic acids; WPs, wall polysaccharides; n.d., not determined. Choline residues are found in WTAs and LTAs.*

### Cell-Surface Proteins Covalently Bound to the Peptidoglycan: The LPXTG- Proteins

Covalent binding of LPXTG-proteins to the CW has been the subject of intensive studies and is certainly one of the best characterised molecular mechanisms for protein anchoring to the PG (Fischetti et al., 1990; Schneewind et al., 1992). Here, we review the major mechanism of anchoring. In parietal monoderm bacteria, a range of proteins called LPXTG (IPR019948) is covalently linked to the PG by enzymes named sortases. Among LPXTG-proteins are found colonising factors, toxins and proteases. In parietal monoderms, the LPXTG motif was identified in both classes of Actinobacteria and Firmicutes, especially in the orders of Coriobacteriales, Streptomycetales, Propionibacteriales, Bifidobacteriales, Micrococcales, and Corynebacteriales for the former, and the orders of Erysipelotrichales, Clostridiales, Lactobacillales, Bacillales, and Tissierellales for the latter. This is a C-terminal motif composed of the LPXTG sequence where X represents any amino acids, followed by a hydrophobic domain and a short positively charged tail. Several variations around this motif were reported, e.g., NP(Q/K)TN, but the overall motifs remain homologous and are included for profile search (Boekhorst et al., 2005). In any case, the motif is recognised by sortases that are classified into six classes from A to F (Dramsi and Bierne, 2017; Siegel et al., 2017). Sortase A anchors a wide range of LPXTG-proteins, whereas sortase B recognises the NP(Q/K)TN related motif. Sortase C allows the pilus assembly (see below), whereas sortases D, E, and F have been much less characterised. Sortases anchor the LPXTG-proteins on the nascent PG through their transpeptidase activity, by cleaving between T and G (or N) and transferring the protein on the PG. Depending on the PG nature, molecular binding can occur at the pentaglycine crossbridge (Marraffini and Schneewind, 2005) or at the A2pm (Budzik et al., 2008).

### Cell-Surface Proteins Non-covalently Bound to the Peptidoglycan

Besides covalent binding to the PG, some proteins exhibit conserved motifs enabling specific binding to the CW components via weak interactions, such as van der Waals interactions, hydrogen or ions bonds.

#### Lysin Motif Domain

LysM (lysin motif) domain was first reported in a protein encoded by gene 15 of *B. subtilis* bacteriophage 364 ϕ29 (Garvey et al., 1986) and exhibiting lysozyme activity that is involved in PG degradation. This conserved domain is found across all kingdoms and is widely distributed among bacteria, although mainly found in Firmicutes, Proteobacteria, Actinobacteria, and Bacteroidetes. A LysM domain (IPR018392) consists of 43–50 amino acids including the first 16 residues that are highly conserved. Multiple LysM domains are often separated by linkers that are rich in S, T, and N residues. From 1 up to 12 LysM domains can be found in a single protein. In bacteria, LysM domains are shown to bind directly the PG in a non-covalent manner (Mesnage et al., 2014). In *E. faecalis*, the LysM domains of AtlA interact with the *N*-acetyl group of the NAG with a minimum of two PG disaccharides NAG-x-NAG (where x corresponds to 1/4 of NAG or NAM). Interestingly, AtlA binds chitin with a higher affinity than PG (Mesnage et al., 2014). This may explain that the presence of a WTA covalently linked at the C6 position of NAM prevents interaction between PG and LysM domains (Steen et al., 2003; Frankel and Schneewind, 2012). Three LysM domains are sufficient for proper binding of AcmA, the major *N*-acetylglucosaminidase of *Lactococcus lactis* (Steen et al., 2005). However, multiple LysM domains are not forming a quaternary structure. In contrast, each LysM domain has a different affinity for the glucide interaction and is thought to bind glycan chains in a cooperative manner (Wong et al., 2014).

#### WXL Domain

The WXL domain (IPR027994) comprises two highly conserved sequence motifs Trp-X-Leu (WXL) including the distal motif YXXX(L/I/V)TWXLXXXP within the last ∼120 to 190 C-terminal of extracellular proteins (Siezen et al., 2006; Brinster et al., 2007). Initially observed in *Lactobacillus plantarum* (Kleerebezem et al., 2003), *Lactobacillus coryniformis* (Schachtsiek et al., 2004), and *Lactobacillus sakei* (Chaillou et al., 2005), extracellular proteins with a C-terminal WXL domain are predicted mainly in the orders of Lactobacillales and Bacillales, such as *B. cereus, Listeria monocytogenes, Lactococcus garvieae, Lactobacillus rhamnosus, Lactobacillus casei* (Siezen et al., 2006; Brinster et al., 2007; Dumas et al., 2008; Morita et al., 2009, 2011; Renier et al., 2012; Toh et al., 2013). The WXL domain was demonstrated to direct proteins to the bacterial cell surface by non-covalent binding to PG (Brinster et al., 2007). Consistently, WXL-proteins localise both at the cell surface and in the culture medium and bind to the surface of parietal monoderm bacteria in *trans* (Schachtsiek et al., 2004; Brinster et al., 2007). Genes encoding WXL-proteins are often organised in clusters (Siezen et al., 2006; Brinster et al., 2007; Galloway-Pena et al., 2015). The hypothesis that proteins of WXL clusters could form multicomponent complexes at the bacterial surface was recently substantiated by the interaction of two *Enterococcus faecium* WXL-proteins with their cognate transmembrane protein *in vitro* (Galloway-Pena et al., 2015). WXL-proteins remain poorly characterised at the experimental level (Siezen et al., 2006; Brinster et al., 2007; Cortes-Perez et al., 2015; Galloway-Pena et al., 2015). Functional, structural, and biochemical analyses of these proteins are urgently required to elucidate their architectural and biological properties.

#### SH3b Domains

SH3 [sarcoma (src) homology-3] domains were first described in eukaryotic proteins. They consist of 60 amino acids in average. In eukaryotes, SH3 domains are mainly involved in protein–protein interactions (Kaneko et al., 2008). In bacteria, SH3-like domains are named SH3b. However, in most articles authors named them indifferently SH3, SH3b, or with other names, e.g., GW. To avoid the promulgation of confusing statements in the scientific literature, they were here named according to the Pfam classification. Accordingly, five subgroups of SH3b domains are reported in parietal monoderm bacteria: SH3 of type 3 (PF08239), SH3 of type 5 (PF08460), SH3 of type 6 (or SH3b1; PF12913), SH3 of type 7 (or SH3b2; PF12914), and SH3 of type 8 (or GW; PF13457). These different SH3 domains allow recognition and binding to PG, but some would also be involved in protein–protein interactions, as suggested for the SH3 domain of type 3 (Rudolf et al., 2015). Of note, the SLAP domain (IPR024968) found in some bacterial cell surface proteins (Boot et al., 1995) may be distantly related to SH3 but further phylogenetic as well as experimental evidences of its implication in CW binding are most required.

The SH3 of type 5 (SH3-5) is a domain of 63 amino acids and is mainly found among Firmicutes, especially *Streptococcus* and *Lactobacillus* genera. This domain described in lysostaphin and Ale-1 proteins binds the pentaglycine peptide bridges of PG (Grundling and Schneewind, 2006; Lu et al., 2006). The SH3 of type 5 could be divided in two subgroups that bind PG with either low or high affinity (Becker et al., 2009).

The SH3 of type 6 and of type 7 were identified in a major class of CW endopeptidases, the NlpC/P60 hydrolases that cleave the linkage between D-Glu and A_2_pm (or K residue; Xu et al., 2015). The SH3 of type 6 (SH3-6) is suggested to bind the crossed-linked stem peptide of the PG. In contrast, SH3 of type 7 does not bind directly the cell surface but may be involved in the interaction between the SH3 of type 6 and the other protein domains (Xu et al., 2015).

The most well studied SH3-like domain is the SH3 of type 8 (SH3-8), also well-known as the GW (Glycine-Tryptophan rich) domain (Braun et al., 1997). In parietal monoderm bacteria, this domain of approximately 80 amino acids is mainly found among Firmicutes, especially *Bacillus, Listeria, Lactobacillus*, and *Staphylococcus* genera. In InlB from *L. monocytogenes*, the SH3 of type 8 was first described to be required for the non-covalent anchoring to the cell surface through an interaction with LTAs (Jonquieres et al., 1999). More recently, however, it was demonstrated to allow non-covalent anchoring directly to the PG (Percy et al., 2016). In the autolysin Atl from *S. epidermidis*, this domain was shown to be responsible for the direct binding to the PG (Biswas et al., 2006), but later, it was proposed to be responsible for the binding to LTAs (Zoll et al., 2010, 2012). Interestingly, in Lactobacilli, this domain is exclusively present in those proteins that harboured a S-layer (Johnson et al., 2015). These domains are also involved in the binding to the host cell receptors, or heparan sulphate proteoglycans (Jonquieres et al., 2001; Marino et al., 2002). They also have been reported to trigger MET phosphorylation and cellular phenotype and to bind Human Thrombospondin 1 and Vitronectin (Bleymuller et al., 2016). Overall, no consensus on the binding ligand for this domain is proposed, which would require further investigations. Nonetheless, a protein with less than two SH3-8 domains cannot bind to the CW (Braun et al., 1997; Jonquieres et al., 1999; Marino et al., 2002; Desvaux et al., 2010; Renier et al., 2012). In some proteins, the designated SH3b domain is not detected by InterPro/Pfam profiles; for instance, and in addition to a choline binding domain CWB1 (cell wall binding repeat of type 1, see below), LytB from *S. pneumoniae* exhibits a SH3b-like domain suggested to be involved in PG recognition (Bai et al., 2014). As this SH3b-like domain does not belong to any of the different types of SH3 domain described above, it suggests that novel types of SH3 domains remain to be uncovered.

#### Sporulation-Related Domain

The sporulation-related (SPOR) domain (IPR007730) was first described in the CwlC of *B. subtilis* (Mishima et al., 2005). CwlC is a CW amidase involved in PG hydrolysis of the mother cell allowing the release of the spore. This hydrolase property led to the name of SPOR domain. A SPOR domain consists of two repeats of 35 amino acid residues; from one to five SPOR domains can be found in a single protein. Among parietal monoderm bacteria, this domain was mainly identified in Firmicutes, especially in Clostridiales and Bacillales. This conserved domain binds the glycan part of PG and binding occurs in a cooperative manner (Mishima et al., 2005). Proteins harbouring a SPOR domain are essentially involved in sporulation or in cell-division processes (Yahashiri et al., 2015, 2017). For example, CwlC is a PG amidase secreted during sporulation and that hydrolyses the mother cell PG. It was proposed that SPOR-proteins, involved in the division process are preferentially localised at the septum, where amidases remove the stem peptides from the PG glycan chains. Thus, protein localization may be due to the binding of SPOR domains on naked PG, i.e., glycan strands lacking stem peptides, which are more abundant at the bacterial septum (Yahashiri et al., 2015).

#### Cell Wall Binding Repeat of Cpl-7

The cell wall binding repeat of Cpl-7 (CW-7; IPR013168) was originally found in the lysin encoded by the *S. pneumoniae* bacteriophage Cp-7 (Bustamante et al., 2010). This domain can be as single or up to three repeats in tandem, essentially in CW hydrolases. CW-7 was further shown to bind specifically to PG, with the CW muropeptide GlcNAc-MurNAc-Ala-isoGln as recognised CW target (Bustamante et al., 2012).

#### Other Domains Involved in PG Non-covalent Binding

Some other domains are described as potentially involved in recognition and non-covalent binding to PG. Among them, many phage lysins targeting the PG of *L. casei* harbour a novel type of PG-binding domain that is highly specific for amidated d-Asp Cross-bridge (Regulski et al., 2013). Other putative domains, mostly found in some Firmicutes, such as PG-binding domain of type 1 (PGB1; IPR002477; Layec et al., 2008) as well as PGB2 (IPR014927) were reported and would require further in-depth characterizations. In *S. pneumoniae*, LytB further exhibits a putative chitin binding domain (WW) domain, which was also proposed to be involved in PG binding (Bai et al., 2014).

### Cell-Surface Proteins Bound to Cell Wall Polysaccharides

#### Cell Wall Binding Repeat of Type 1

The CWB1 (IPR018337) is also called choline-binding (ChBD) or CW binding repeat; for clarity and in echo to the cell wall binding repeat of type 2 (CWB2) reviewed here below, the CWB1 is preferred and favoured. This conserved domain is approximately 20 amino acids long. In parietal monoderm bacteria, CWB1 is mainly found among Firmicutes, especially in the families of the Lachnospiraceae, Ruminococcaceae, Clostridiaceae, Lactobacillaceae, and Streptococcaceae but also in some Actinobacteria, e.g., the Coriobacteriia and Bifidobacteriales orders. It was hypothesised that *S. pneumoniae* possessed an autolysin able to interact with phosphatidyl choline residues of the WTAs (Holtje and Tomasz, 1975). More than 10 years later, a glycosyltransferase, able to bind WPs through a repeated unit of amino acids was reported in *Streptococcus sobrinus* (Ferretti et al., 1987). This report was just followed by the demonstration that similar repeats in the lytic proteins of *S. pneumoniae* phage were involved in the recognition of choline-containing CWs (Garcia et al., 1998). Several surface proteins, including LytA from *S. pneumoniae*, were described to possess such a domain that was named glucan-binding domains (GBDs) and eventually CWB1 (Giffard and Jacques, 1994). LytA was the most characterised enzyme because it mediates indirectly virulence by lysis, allowing the release of toxins. The four LytA CWB1 domains were co-crystallised with choline (Fernandez-Tornero et al., 2001). Four choline interacting CWB1 sites are found in LytA, implying that at least three CWB1 are needed to form an interaction with one molecule of choline. It was then suggested that proteins harbouring less than three CWB1 are not expected to have affinity for CW choline residues.

#### Cell Wall Binding Repeat of Type 2

The CWB2 domain (IPR007253) was identified in CwlB of *B. subtilis* (Kuroda and Sekiguchi, 1991). In this species, CwlB is the major amidase. The CWB2 domain is approximately 90 amino acids long. In parietal monoderm bacteria, the CWB2 domain is found in the class of Actinobacteria, especially in the Micrococcales order, and the class of Firmicutes, especially in the orders of Clostridiales and Bacillales. Most of proteins carrying the CWB2 domains are reported with triple adjacent domains, more rarely with one or two (Fagan et al., 2011), e.g., the 29 Cwps (CW proteins) of *Clostridium difficile* all harboured three CWB2 domains. Among them, SlpA is the main S-layer protein of the *C. difficile*. Other Cwps were assigned with different potential functions, including amidase and protease (Fagan et al., 2011). This organisation may be due to the three-dimensional architecture; the formation of CWB2 trimer was indeed proposed to interact with the CW via a non-covalent linkage with the polysaccharide II (PSII; Willing et al., 2015). In *C. difficile*, the PSII is covalently anchored via a phosphodiester bond to the PG. In Cwp6 (CW protein 6) and Cwp8 from *C. difficile*, the trimer structure was recently solved by crystallography (Usenik et al., 2017). This structure revealed that 12 conserved residues were located between two domain interfaces. Moreover, using docking experiments, the structure formed by the CWB2 trimer was confirmed to be compatible with an interaction with the six monosaccharides that composed the PSII (Usenik et al., 2017). Two conserved surface R residues that may interact with the PSII are also found in the S-layer homology (SLH) trimers (see below; Kern et al., 2011). This result in combination with the SLH organisation in trimer and a similar function of polysaccharide anchoring suggests a common or convergent evolutionary origin (Kern et al., 2011).

#### S-Layer Homology Motif

The SLH domain (IPR001119) was first reported in three proteins of *Clostridium thermocellum* (Fujino et al., 1993). This domain was later named SLH after comparison of the S-layer protein sequences of *Acetogenium kivui, C. thermocellum*, and *Bacillus brevis* (Ebisu et al., 1990; Lupas et al., 1994). This domain consists of an approximately 55-amino acid-long sequence with a group of five highly conserved residues (ITRAE). In parietal monoderm bacteria, it is identified in some species of the class Actinobacteria, such as in the order Coriobacteriales, Bifidobacteriales, or Micrococcales, but mainly among Firmicutes, including Clostridia and Bacilli. Three SLH domains were shown to be sufficient for the anchoring at the CW surface of *B. anthracis*, but only two are necessary for the CW interaction (Mesnage et al., 1999; Huber et al., 2005). Moreover, SLH proteins from *C. thermocellum* are able to bind the CW of *B. anthracis* and *vice versa* (Chauvaux et al., 1999). SLH domains are shown to bind WP in a non-covalent manner. The WP fraction is pyruvylated by CsaB and this WP modification is essential for the SLH protein binding (Mesnage et al., 2000). The Sap structure, a *B. anthracis* S-layer protein, confirmed the potential interaction between WP and the three SLH motifs (Kern et al., 2011). Pyruvate was later found to be placed at the distal end of each WP (Forsberg et al., 2012). In *Bacillus*, it is proposed that the *N*-acetyl mannose of the WP is pyruvylated (Forsberg et al., 2012). In some cases, the SLH domains may be not sufficient for WP interaction. For instance, in the SbsB of *Bacillus sphaericus*, the C-terminal domain together with the SLH domains is needed for the WP interaction (Huber et al., 2005). Direct and exclusive binding of SLH to the PG or together with the WP is still subject to discussion and remains controversial (Zhao et al., 2005, 2006; Janesch et al., 2013).

### Cell-Surface Proteins Bound to the CW by Unknown Mechanism

The ChW (clostridial hydrophobic repeat with a conserved W residue) domain (IPR006637) was first identified in *Clostridium acetobutylicum* and was predicted to be involved in cell surface anchoring or in protein–protein interaction (Nölling et al., 2001; Desvaux, 2005a). This domain is constituted of highly conserved GW dipeptide motifs and is about 50 amino acids long. A single protein can harbour between one and 12 ChW domains. It was suggested that the ChW domains are associated in triplet for the surface interaction but the biochemical nature of the CW ligand remains unknown (Sullivan et al., 2007). In parietal monoderm bacteria, the ChW domain is essentially found in some Firmicutes, especially of the class Clostridia, but also in some Erysipelotrichia and Bacilli, e.g., in the genera *Lactococcus, Streptococcus*, and *Enterococcus*, as well as in some bacteria of the phyla Actinobacteria, especially in the genus *Streptomycetes*. ChW-proteins are mostly endolysins suggesting the importance of this domain for CW interaction and enzymatic activity (Oliveira et al., 2013).

### Cell-Surface Proteins with Uncharacterised Cell-Envelope Interacting Domain: The Moonlighting Proteins

Parietal monoderm bacteria have some surface-exposed proteins that lack a canonical signal sequence and a CW interacting domain. Although not sharing any domain or sequence homology, they share the ability to interact with fibronectin or extracellular matrix (ECM)-components. The most common are cytoplasmic enzymes or proteins, referred as moonlighting proteins. They include the ubiquitous glycolysis enzymes glyceraldehyde-3-phosphate dehydrogenase (GAPDH; Pancholi and Fischetti, 1992), enolase, phosphoglycerate kinase, the glutamine synthetase (GlnS), and the translation elongation factor Ef-Tu (Amblee and Jeffery, 2015). While most act as adhesins by interacting with components of the host ECM (plasminogen, fibronectin, laminin, or mucin), some like Ef-Tu interact also with neuropeptides at the membrane level (Fulde et al., 2013; Mijouin et al., 2013; Amblee and Jeffery, 2015; N’Diaye et al., 2016a,b, 2017). Beside the anticipated lack of an SP and cell surface association domain, bioinformatic analysis of 98 experimentally reported intracellular proteins having a moonlighting cell surface function, failed to identify specific features shared by these proteins (Amblee and Jeffery, 2015). The domain interacting with plasminogen is frequently localised at the C-terminus of the protein, however, no conserved domain could be identified (Bergmann et al., 2003; Ehinger et al., 2004). Ionic bonds and low pH were shown to contribute to the association of cytoplasmic proteins with the cell surface of *Lactobacillus crispatus* (Antikainen et al., 2007). Conversely, GAPDH and enolase have been shown to bind LTA on the bacterial cell surface by ionic bonds (Antikainen et al., 2007; Kinoshita et al., 2008). Reversely, GAPDH of *S. pneumoniae* did not bind synthetic LTAs or TAs and direct binding to PG was observed (Terrasse et al., 2015). Moonlighting proteins occur in all bacteria and are thus involved in a large range of unrelated functions including colonisation, modulation of the host response and virulence (Kainulainen and Korhonen, 2014).

Other non-classical proteins exposed on the cell surface of parietal monoderm bacteria are known as fibronectin-binding proteins (FBPs) characterised by two adjacent conserved domains: the about first 400 amino acids (PF05833) of which 89 residues associate with fibronectin-binding activity (Courtney et al., 1994) followed by the conserved domain of unknown function DUF814 (IPR008532) of ∼100 amino acid residues including conserved motif (D/E)X(W/Y)XH. First identified in the fibronectin-binding protein FBP54 of *S. pyogenes* (Courtney et al., 1994), these domains have been reported in PavA of *S. pneumoniae* (Holmes et al., 2001), FbpA of *Streptococcus gordonii* (Christie et al., 2002), Fbp68 of *C. difficile* (Hennequin et al., 2003), FbpB of *Clostridium perfringens* (Katayama et al., 2009), EfbA in *E. faecalis* (Torelli et al., 2012), Fnm in *E. faecium* (Somarajan et al., 2015), YloA in *B. subtilis* (Rodriguez Ayala et al., 2017) and FbpA in *Weissella cibaria* (Wang et al., 2017). Consistently with impaired binding capacity to fibronectin upon nested deletions of the C-terminal part of *S. pneumoniae* PavA (Holmes et al., 2001), structural and functional analyses of FBPS of *Streptococcus suis* revealed that the C-terminal half of FBPs mediates binding to fibronectin whereas the N-terminal half interacts specifically with the surface of *Streptococcus suis* (Musyoki et al., 2016). The fact that N-terminal half of FBPs does not bind to *S. pneumoniae* nor *S. agalactiae* cells suggests an interaction with a specific CW component that remains to be identified. Despite their contribution to fibronectin binding and overall virulence in several pathogens, the exact role of these FBPs is still unclear (Kawabata et al., 2001; Dramsi et al., 2004; Pracht et al., 2005; Torelli et al., 2012; Somarajan et al., 2015).

How these proteins are released outside from the cell and attached to the cell surface remains poorly understood. Several lines of evidence indicate that the release of GAPDH of *S. agalactiae, S. aureus*, and *S. pneumoniae* bacterial involves autolysis (Pasztor et al., 2010; Oliveira et al., 2012; Terrasse et al., 2015). Consistently, moonlighting proteins localise preferentially at the septum. However, this issue is still debated as not all cytoplasmic proteins are detected at the CW (Ebner et al., 2016). Interestingly, based on indirect evidence using an inhibitor of a mechanosensitive channel it was recently proposed that EF-Tu and DnaK of *S. epidermidis* could be exported through the large mechanosensitive channel (N’Diaye et al., 2016b). Variations of channel diameter between bacterial species may explain differences between patterns of moonlighting proteins.

## Cell-Surface Supramolecular Protein Structures

Besides monopolypeptides, some surface proteins form complex surface organelles. In parietal monoderm bacteria, such supramolecular protein structures include the S-layer, flagellum, various pili and cellulosome.

### S-Layer

S-layer is a proteinaceous two-dimensional crystalline array constituting the outermost CW layer in the absence of a capsule (Fagan and Fairweather, 2014). Located above the PG, this surface supramolecular structure is not a common theme in parietal monoderm bacteria, e.g., it is present in numerous *Bacillus* or *Clostridium* species but absent from *Listeria* and *Staphylococcus* genera. Usually, a S-layer is formed by the auto-assembly of a unique protein that may be glycosylated. The S-layer proteins are usually rich in hydrophobic and acidic amino acids (Sára and Sleytr, 2000). The interactions between the S-layer subunits are stronger than surface interactions (Messner and Sleytr, 1992). Most often bacteria with an S-layer possess a single S-layer and in very rare cases two (Kuen et al., 1997). Most of the S-layer proteins are non-covalently anchored through SLH or CWP domains at the bacterial surface. Of note and as mentioned above, a protein harbouring a SLH or a CWP domain is not necessarily an S-layer protein.

The function of the S-layer remains unclear but it is generally suggested to act either as a scaffold, a sieve or a shield to some environmental stresses (Sára and Sleytr, 2000; Fagan and Fairweather, 2014; Gerbino et al., 2015). While cited as a virulent factor or adhesion factor, such a role has not been convincingly demonstrated in any parietal monoderm bacteria. In *B. anthracis*, Sap was suggested to be the receptor of the phage AP50c (Plaut et al., 2014). Investigating its contribution to colonisation processes, S-layer was negatively correlated with biofilm formation in *B. cereus* (Auger et al., 2009). Consistently, a *C. difficile* mutant strain lacking the Cwp84 protease, which plays a key role in the maturation of the S-layer protein SlpA, forms a biofilm 72-fold more important than the wild type strain (Pantaleon et al., 2015). Except for *C. difficile* (Merrigan et al., 2013), the bacterial S-layer is considered as non-essential. The S-layer proteins can account for up to 15% of total bacterial proteins, also the absence of common physiological functions among bacteria is intriguing. Undoubtedly, this call for in-depth investigations under conditions more relevant to the ecophysiology of the bacterial species considered.

### Flagellum

The bacterial flagellum is secreted and assembled via the FEA. Several transmembrane components constitute the translocon (FlhAB-FliOPQR) and form the translocase together with the ATPase FliI (Macnab, 2003, 2004). The flagella *per se* is composed of a basal body, the hook, the junction and the filament proteins, which are secreted and assembled by the FEA (Evans et al., 2014). These proteins do not exhibit a SP and the signal necessary for targeting is still controversial (Aldridge and Hughes, 2001, 2002). While most knowledge about the assembly and regulation mechanisms results from investigations in different LPS-diderm bacteria, information related to parietal monoderm bacteria remains restricted to fewer bacterial species, e.g., *B. subtilis* (Mukherjee and Kearns, 2014; Rossez et al., 2015).

Of course the flagellum is a well-known motility factor that can be involved in swimming but also swarming (Henrichsen, 1972; Belas, 2014). Swarming is especially relevant for surface colonisation processes, including adhesion and biofilm formation (Beeby, 2015; Chaban et al., 2015). Mechanosensing by flagella and chemotaxis further allow the bacteria to switch developmental programmes and adapt in response to changes in their environment. Glycosylation of the flagella has now been demonstrated in several parietal monoderm bacteria (Schirm et al., 2004, 2005; Twine et al., 2008, 2009; Kajikawa et al., 2016) and they further appeared to play a role in pathogenesis and biofilm formation (Valguarnera et al., 2016; Valiente et al., 2016).

### Pili

Pili are tubular cell-surface appendages, which size, diameter, and shape can be extremely variable depending on the type of appendage considered. In parietal monoderm bacteria, three main types of pili can be encountered, (i) the pili made of covalently linked subunits involved in colonisation and host interaction, (ii) the type 4 pili (T4P) involved in transformation, motility and adherence, and the most recently uncovered (iii) pili made of amyloids. In parietal monoderm bacteria, beside the evidence the formation of a DNA translocation channel, no conjugative pili has been formally demonstrated.

#### Covalently Assembled Pili

First reported and studied in the mycolic-diderm Actinobacteria and Corynebacteria, pili made of covalently linked pilins are assembled and anchored to the PG by sortases (Yanagawa et al., 1968; Ton-That and Schneewind, 2003). Since then they have been described in various parietal monoderm bacteria, e.g., including some bacilli, enterococci, streptococci, lactococci, lactobacilli, and bifidobacteria (Ton-That and Schneewind, 2003; Kankainen et al., 2009; Hendrickx et al., 2011; O’Connell Motherway et al., 2011; Oxaran et al., 2012; Murphy et al., 2014). Pili are all composed of a major pilin that forms the shaft and a minor tip pilin that is located at the tip of the pilus. Genes encoding pili are organised in operon of two or three prepilin genes and one or two pilin-specific sortase enzymes (Hendrickx et al., 2011). All prepilins contain an N-terminal SP for secretion and a C-terminal LPXTG domain for covalent binding to PG or formation of intermolecular bonds between pilins. In addition, they exhibit tandem Ig-like domains, also referred as CnaB domains (PF16569), contributing to pili integrity, stability, and biomechanical properties through self-generated intramolecular bonds between a lysine and an asparagine residue (Kang et al., 2007; Budzik et al., 2009; Kang and Baker, 2012; Echelman et al., 2016). The YPNK motif is typical of major and basal pilins and provides the K residue to form the intermolecular isopeptide bond with another molecule of pilin. Besides, major pilins have a conserved glutamate residue in an E-box motif (consensus YXLXETXAPXGY) that contributes to the autocatalytic formation of intramolecular isopeptide bonds (Budzik et al., 2009; Kang et al., 2009; Alegre-Cebollada et al., 2010). Basal pilins are usually smaller and have a proline-rich C-terminal tail involved in CW anchoring (Krishnan et al., 2007; Linke et al., 2010). Pilins are assembled by sequential transpeptidation reactions involving sortases. Successively, the threonine of the LPXTG sorting signal of the minor tip pilin is covalently linked to the conserved K residue of the YPKN pilin motif of the major pilin by a pilus-specific class C sortase (Budzik et al., 2008). Subunits of the major pilin are then successively polymerised head-to-tail by the pilus-specific sortase. High resolution transmission electron microscopy and pilin structural studies confirmed that these pili were heteropolymers of two to three head-to-tail covalently linked pilins (Kang et al., 2009). Once assembled and depending on the species, the pilus is generally anchored to the PG by the housekeeping sortase A either directly or through the incorporation of the minor basal pilin (Dramsi et al., 2006; Budzik et al., 2007; Mandlik et al., 2008; Necchi et al., 2011; Shaik et al., 2014). Tip pilins do not exhibit YPKN motif, but consistently with their adhesive function they harbour adhesion domains, e.g., vWFA (von Willebrand factor A) domain, in addition to classical IgG-like folds (Krishnan et al., 2007; Linke et al., 2010). However, several exceptions to this general picture have been reported, e.g., the pilin motif YPKN can be restrain to a single lysine (Kang et al., 2007; Cozzi et al., 2015), tip and basal pilins can spread along the pilus shaft (Dramsi et al., 2006; Kankainen et al., 2009; Reunanen et al., 2012; Yu et al., 2015), sortase A can be dispensable for pilus anchoring to the CW (LeMieux et al., 2008; Lazzarin et al., 2015).

Covalently assembled pili are essentially involved in colonisation processes, especially sessile development (Nallapareddy et al., 2006; Krishnan et al., 2007; Manetti et al., 2007; Konto-Ghiorghi et al., 2009; Pointon et al., 2010; Rinaudo et al., 2010; Sillanpaa et al., 2010, 2013; Danne et al., 2011; Oxaran et al., 2012). Zipper-like interactions favoured by multiple SpaC distributed along the pilus were suggested a major contributor to biofilm formation (Tripathi et al., 2013). These pili can also play key roles in bacterial adhesion to ECM proteins, e.g., fibronectin, collagens or mucins, via the tip pilin (Schwarz-Linek et al., 2003; Hilleringmann et al., 2008; von Ossowski et al., 2011). Covalent intra- and intermolecular bonds of covalently assembled pili confer remarkable spring-like biomechanical properties, which can withstand physiological shear forces. In addition to specific heterophilic interactions with mucin and collagen, tip pilin SpaC mediates homophilic interactions involved in bacterial aggregation (Tripathi et al., 2013). In *S. pyogenes*, the N-terminal thioester domain of the pilus adhesin Cpa was demonstrated to form covalent bonds with the polyamine spermidine, suggesting these pili could be involved in covalent attachment to host cells (Linke-Winnebeck et al., 2014).

#### Non-covalently Assembled Pili: The Type 4 Pili

Initially described and thoroughly studied in LPS-diderm bacteria, type 4 pili (T4P) are thin flexible filaments (5–8 nm) of several microns in length composed of thousands of copies of a major pilin (Craig et al., 2003). T4P pili are helical polymers of a major, which consists in a conserved α-helix at the N-terminus followed by a C-terminal β-sheet domain (Craig et al., 2004). The cohesion of the filament relies on hydrophobic interactions between the N-terminal helices amino acids N-terminal α-helices in the centre of the fibre. The presence of filaments resembling T4P in parietal monoderm bacteria was first observed in *Ruminococcus albus* (Rakotoarivonina et al., 2002). Since then, clusters of genes associated to T4P formation, have been detected in many genomes of Firmicutes (Imam et al., 2011; Berry and Pelicic, 2015). In parietal monoderm bacteria, components of the T4P are secreted and assembled by the FPE. According to *B. subtilis* nomenclature, the FPE system is composed of the ATPase ComGA, the IMP ComGB and the type 4 prepilin peptidase ComC, whereas ComGC is the major pilin, ComGD, ComGE, ComGF, and ComGG are minor pilins (Chen et al., 2006; Desvaux and Hébraud, 2006, 2009). In *C. perfringens* and *S. sanguinis*, the FPE comprises a retraction ATPase (IPR006321) and two conserved proteins involved in pili assembly (IPR005883 and IPR007813) in addition to the assembly ATPase ComGA (IPR001482), the IMP ComGB (IPR003004) and the type 4 prepilin peptidase ComC (IPR000045) (Melville and Craig, 2013; Berry and Pelicic, 2015). Besides, the T4P is composed of two major and three minor pilins (IPR012902). Based on models derived from LPS-diderm bacteria where the T4P is secreted and assembled by a type II secretion system (Peabody et al., 2003; Desvaux et al., 2009; Chagnot et al., 2013), the prepilins of <200 amino acid residues exhibit a SP with a conserved type 4 prepilin motif including a glutamate at position 5 of the mature protein (Tjalsma et al., 2000; Desvaux and Hébraud, 2006, 2009). Prepilins are processed by the prepilin peptidase cleaving the SP between the n- and h-domain. Polymerization of the mature pilins involves the assembly ATPase and integral membrane and accessory proteins. When present, the retraction ATPase mediates depolymerization of pilin subunits and subsequent pilus retraction (Melville and Craig, 2013; Berry and Pelicic, 2015). Structural characterization of PilA1, the major pilin of the T4P in *C. difficile*, confirms general structural conservation with an N-terminal α-helix, followed by a helical αβ-loop and a four-stranded anti-parallel β sheet, instead of the typical the C-terminal disulfide bond of type 4 pilins (Piepenbrink et al., 2015). Amino acid sequence variation in the C-terminal part of PilA1 between strains revealed alternative stabilising hydrogen bonds between the β loops, highlighting that T4P of parietal monoderms rely on specific mechanisms in spite structural and function conservation with those of LPS-diderm bacteria. Interestingly, *S. sanguinis* encodes an additional T4P, which proteins are orthologous to proteins involved in the assembly of the T4P in *B. subtilis* and *S. pneumoniae* (Xu et al., 2007; Gurung et al., 2016; Gurung et al., 2017).

T4P are generally involved in twitching motility, DNA uptake during conjugation and transformation, adherence to host cells and biofilm formation (Giltner et al., 2012). In parietal monoderm bacteria, the involvement of T4P in bacterial motility have been reported in *C. perfringens* and *S. sanguinis* (Varga et al., 2006; Gurung et al., 2016), whereas transformation by T4P has been experimentally demonstrated in *S. pneumoniae* (Laurenceau et al., 2013, 2015). It was evidenced that DNA fragments are too large to go through the T4P and exogenous double-stranded DNA would actually be captured by the pilus before being guided to the Com (competence development) uptake machinery (Dubnau, 1999; Dubnau and Provvedi, 2000; Chen and Dubnau, 2004; Johnston et al., 2014; Laurenceau et al., 2015). In *R. albus*, T4P is specifically involved in adherence to cellulose (Rakotoarivonina et al., 2002).

#### Amyloid Pili

Among macromolecular structures displayed at the surface of parietal monoderm bacteria, amyloid pili remain poorly characterised. Reminiscent of the curli in Enterobacteriaceae, these amyloid fibres are quaternary structure of peptide or protein aggregates forming parallel β-sheets perpendicular to the fibre axis (Rambaran and Serpell, 2008). Initially reported in *B. subtilis*, amyloid pili have so far mainly been involved in biofilm formation (Taglialegna et al., 2016a). Their biogenesis relies on different steps depending on the precursor protein, but always leads to stable β-sheet aggregates. Secreted by the Sec pathway, the *B. subtilis* amyloid protein TasA forms amyloid fibres of variable length and 10–15 nm in width (Romero et al., 2010). The co-encoded dedicated signal peptidase SipW and TapA are required to process and produce functional TasA fibres, respectively (Romero et al., 2011, 2014). Like for other amyloidogenic precursors, acidic pH promotes aggregation of TasA (Chai et al., 2013). However, the mechanism of TasA amyloïd fibre biogenesis is still unknown. In *S. aureus*, amyloids fibres are made of secreted peptides known as phenol-soluble modulins (Schwartz et al., 2012, 2014; Marinelli et al., 2016; Tayeb-Fligelman et al., 2017). The LPXTG-cell wall anchored adhesins P1 (AgII) and WapA in *Streptococcus mutans* and BapA in *S. aureus* have the ability to form amyloid fibres (Oli et al., 2012; Taglialegna et al., 2016b; Besingi et al., 2017). BapA is a member of biofilm-associated protein (Bap) family defined as high-molecular-weight CW anchored LPXTG proteins involved in biofilm formation (Shankar et al., 1999; Cucarella et al., 2001; Lembre et al., 2014). In BapA, the domain B self-assembles into amyloid fibres in acidic pH and low calcium concentration (Taglialegna et al., 2016b). Although domain B of BapA orthologue of *Staphylococcus saprophyticus* is amyloidogenic, other peptides may be involved in the biogenesis of Bap amyloid fibres since a short amyloidogenic peptide derived from the imperfect tandem repeats (C-repeats) in Bap proteins of other *Staphylococcus* species has been identified (Lembre et al., 2014). The amyloidogenic moiety is located in the C-terminal region of P1 and central part for WapA (Besingi et al., 2017). The amyloidogenic moiety of P1 also associates with covalently attached full-length P1 suggesting that P1 may serve as the platform for amyloidogenesis (Heim et al., 2014). Although much less characterised, the amyloidogenic moiety of WapA encompasses a collagen-binding domain (IPR008456) composed of two antiparallel β-sheets and two short α-helices. Occurring in specific conditions, amyloidogenesis can be viewed as a conformational adaptation of cell surface proteins with dual functions.

#### Conjugative Pili

Consistently with their ability to exchange DNA, several species of parietal monoderm bacteria have in their genomes mobile genetic elements that encode conjugative systems reminiscent of type IV secretion system (T4SS) in LPS-diderm bacteria (Guglielmini et al., 2013, 2014). Although incompletely understood, the best characterised conjugative elements in Firmicutes are the transposon Tn916 of *E. faecalis*, the plasmids pIP501 of *S. agalactiae*, pCF10 of *E. faecalis* and pCW3 of *C. perfringens*, the integrative and conjugative elements ICEBs1 of *B. subtilis* and ICESt1 of *Streptococcus thermophilus* (Alvarez-Martinez and Christie, 2009; Bhatty et al., 2013; Goessweiner-Mohr et al., 2013; Bellanger et al., 2014; Auchtung et al., 2016; Wisniewski and Rood, 2017). In contrast to conjugation in LPS-diderm bacteria, conjugation in parietal monoderm bacteria would not require pili formation (Andrup and Andersen, 1999). However, considering the identification of several proteins homologous to the T4SS and the analogous situation with the FPE in *B. subtilis* where only pseudo-pilus would be formed in parietal monoderm bacteria, much deeper investigations in that direction would be required to categorically exclude the formation of conjugative pili in any parietal monoderm bacteria. Not to forget that just a couple of decades ago, the presence of any pili in parietal monoderm bacteria was not even considered.

### Cellulosome

A cellulosome is a supramolecular multienzymatic complex present at the bacterial surface of some parietal monoderm bacteria and dedicated to degradation of plant CW polysaccharides (Bayer et al., 2004; Fontes and Gilbert, 2010). It is organised around a scaffolding which assembles different catalytic subunits. Cellulosomes are only found in some parietal monoderm bacteria of the families Lachnospiraceae and Clostridiaceae (Guedon et al., 2000; Desvaux and Petitdemange, 2001; Desvaux, 2005a). In *C. thermocellum*, the scaffolding CipA (cellulosome-integrating protein A) is composed of a CBM (carbohydrate-binding module), a DocII (dockerin domain of type II), and 9 CohI (cohesin domains of type I) (Béguin and Lemaire, 1996; Smith and Bayer, 2013). Whereas different types of CBM allows binding to different carbohydrate-polymers with various affinities, CohI acts as a receptor domain for a dockerin domain of type I (DocI) harboured by cellulosomal enzymes. CipA can display up to nine different cellulosomal enzymes thanks to the 9 CohI and its anchor to the bacterial cell surface via DocII, which interacts with a CohII (type II cohesin domain) presents in a cell-surface protein, such as SdbA (scaffolding dockerin binding A) (Stern et al., 2016). Depending on the bacterial species, the scaffolding can harbour more or less cohesion domains (Schwarz, 2001). An even higher level of complexity can even be reached when different scaffoldings assemble one with another from complex cellulosomes like in *Pseudobacteroides cellulosolvens* or *Acetivibrio cellulolyticus* (Xu et al., 2003, 2004; Hamberg et al., 2014). The assembly of several cellulosomes to form a polycellulosome would occur via DocI/CohI interactions (Carvalho et al., 2003). Cellulosome components exhibit SP and would be secreted by the Sec pathway, even so the mechanism for the assembly of the different subunits at the cell surface appears quite complex (Desvaux, 2005a,b; Bras et al., 2016; Bule et al., 2017; Smith et al., 2017). The cellulosome is generally exposed at the bacterial cell surface following cohesin-dockerin interaction with cell-surface proteins, themselves anchored in the CW via SLH domains.

## Conclusion

By reviewing the components of the cell envelope of parietal monoderm bacteria, this review stressed the difference between the surfaceome, i.e., the totality of molecules present at the bacterial cell surface, and the proteosurfaceome, the proteinaceous subset of the surfaceome. Besides, the concept of bacterial cell surface must be carefully balanced and considered with regards of the idea of scale and the notions of molecular, accessible, contact and reentrant surfaces. Considering both the CM and CW as well as monopolypeptides and supramolecular protein structures, this review provides an overview of the mechanisms of protein anchoring to the cell envelope of parietal monoderm bacteria. Nonetheless, it can hardly be considered as exhaustive. Indeed, some additional domains have been described in some bacterial species but have not been registered in InterPro as yet and/or would demand further characterization. For instance, the C-terminal WrY domain of Sbi (second binding protein for immunoglobulins) from *S. aureus* binds the LTAs (Smith et al., 2012). In Aap (accumulation associated proteins) from *S. aureus*, bioinformatic analyses strongly suggest the G5 domain could be involved in NAG binding but experimental evidences supporting this function are still awaited (Bateman et al., 2005). This is also the case for several S-layer proteins, which do not harbour SLH domains but are clearly attached to the CW (Chami et al., 1997; Schäffer et al., 1999; Steindl et al., 2002; Avall-Jaaskelainen and Palva, 2005). At the opposite, some domains reported in InterPro would still require further characterization since their first report to ascertain their involvement in protein attachment to the CW, e.g., the PGB2 (IPR014927) (Foster, 1991). Of note, no protein domain involved in the interaction with components of the capsule, such as the polyglutamate, has been uncovered so far. Bacteriophages often use cell surface polysaccharides as receptors. Exploration of the diversity of receptor-binding proteins of bacteriophages may help to identify novel WP binding domains although their multimeric state required for efficient binding will imply structural analyses (Regulski et al., 2013; McCabe et al., 2015; Koc et al., 2016).

It can also be stressed that some cell-surface proteins can exhibit several anchoring domains, e.g., some IMPs can also be lipoproteins or some LPXTG-proteins can also have additional CWBDs. Even for the well-characterised domains presented in this review, there is still some work to be done to refine their biochemical properties, especially to decipher in detail the interactions of a given domain with its CW ligand(s) and define their three-dimensional structure, which have been resolved only in a handful of them. For more complex structures, e.g., pili, tomography by cryo-EM is certainly one of the method of choice to reveal their molecular details (Li and Thanassi, 2009). While considered rare or even absent a decade ago, it appears bacterial proteins can be glycosylated, especially those cell-surface exposed (Lu et al., 2015; Schaffer and Messner, 2017). However, the extent to which glycosylation contributes to protein anchoring to the cell envelope of parietal monoderm bacteria remains an open question. All-in-all, it can be bet that novel cell-envelope binding domains will be uncovered in the years to comes and increase the repertoire of known surface proteins in parietal monoderm bacteria. Most of the protein domains interact non-covalently with the CW. Consistently with bacterial growth, the surfaceome is extremely dynamic and in constant renewing. Together with other processes, labile interactions participate to the flexibility and the spatio-temporal remodelling of the surfaceome in response to physiological or environmental changes (Bierne and Dramsi, 2012; Mitra et al., 2016). Undoubtedly, the composition of proteosurfaceome is of great importance for the colonisation of various environments, including bacterial adhesion and biofilm formation ability of parietal monoderm bacteria (Planchon et al., 2009; Renier et al., 2011; Chagnot et al., 2012, 2013).

By providing a comprehensive view of mechanisms of protein anchoring to the cell envelope of parietal monoderm bacteria, this review should be helpful for scientists and researchers involved in global approaches, especially genomics, transcriptomics and proteomics. Predicting the subcellular localization of genome encoded proteins is a key step in comprehending the physiology of a given micro-organism but also applied research dedicated to the mining of new degradative enzymes, adhesins or antigens. Respective to the proteosurfaceome, several individual tools allow predicting the presence of SP, e.g., SignalP (Nielsen, 2017), cell-envelope anchoring domain, e.g., InterProScan (Jones et al., 2014), or even the final subcellular location, e.g., PSORT (Peabody et al., 2016), but an integrated and combining approach based on the secretome concept, which considers the biology of protein secretion by including the protein secretion systems, post-translational and post-translocational modifications as well as retention signals, proved much more powerful than individual predictors (Renier et al., 2012). While genomics is useful for initial mapping of the secretome, which further allows defining the proteosurfaceome (i.e., the cell surface complement of the secretome), proteomics remains the ultimate method of choice to ascertain that proteins are effectively expressed and located as predicted (Planchon et al., 2007; Solis and Cordwell, 2011, 2016). Besides proteomics, which by definition focuses on the whole protein content, glycomics and lipidomics can also be used to investigate globally the polysaccharidic and lipid fractions of the bacterial surfaceome (Chessa et al., 2008; Kondakova et al., 2015). So far, however, such approaches have not been broadly applied in parietal monoderm bacteria but it is certainly a promise in the years to come to define more comprehensively the surfaceome of these bacteria, which are for some of them important pathogens. As a primary target, such research directions on the surfaceome are a prerequisite for the development of novel antibacterial agents or therapeutics.

## Author Contributions

All authors listed have made a substantial, direct and intellectual contribution to the work, and approved it for publication.

## Conflict of Interest Statement

The authors declare that the research was conducted in the absence of any commercial or financial relationships that could be construed as a potential conflict of interest.
